# Using Whole-genome Sequencing to Determine the Timing of Secondary Tuberculosis in British Columbia, Canada

**DOI:** 10.1093/cid/ciaa1224

**Published:** 2020-08-19

**Authors:** Kamila Romanowski, Benjamin Sobkowiak, Jennifer L Guthrie, Victoria J Cook, Jennifer L Gardy, James C Johnston

**Affiliations:** 1 British Columbia Centre for Disease Control, Vancouver, Canada; 2 University of British Columbia, Vancouver, Canada

**Keywords:** tuberculosis, epidemiology, whole-genome sequencing

## Abstract

Combined with epidemiological data, whole-genome sequencing (WGS) can help better resolve individual tuberculosis (TB) transmission events to a degree not possible with traditional genotyping. We combine WGS data with patient-level data to calculate the timing of secondary TB among contacts of people diagnosed with active TB in British Columbia, Canada.

The End TB strategy aims to end the global tuberculosis (TB) epidemic, with targets to cut the global incidence of TB by 90% between 2015 and 2035 [1]. A key component of this strategy is the early diagnosis of TB and the systematic screening of contacts and other high-risk groups (Pillar 1-a) [1, 2]. Contact investigation, an effective systematic screening strategy, is used to identify individuals who have been exposed to infectious TB and ensure people are appropriately assessed for risk of disease through interview and screening (i.e., chest X-ray, tuberculin skin test), and when indicated, treated for active or latent TB [3]. Although estimates exist on the proportion of TB transmission that can be attributed to contacts, particularly household contacts, the time from exposure to the development of active TB is less clear [4].

Genotyping techniques, such as Mycobacterial Interspersed Repetitive Units–Variable Number of Tandem Repeats (MIRU-VNTR), can provide support for potential transmission between source–contact dyads by examining variation in specific regions of the *Mycobacterium tuberculosis* (*Mtb*) genome to identify isolates with the same genotype, suggestive of transmission. However, studies have shown that genotyping techniques can overpredict clustering in specific TB lineages, particularly in low-incidence settings, where MIRU-VNT Rclustering often identifies common community strains rather than recent transmission [5]. In contrast, whole-genome sequencing (WGS) may better resolve individual transmission events to a degree not possible with traditional genotyping [6], improving our understanding of the timing of TB development, postexposure [7]. Thus, for this study, we linked WGS data with patient-level clinical and epidemiological data to identify potential secondary TB transmission among contacts of people with active disease in British Columbia (BC), Canada, between 2005 and 2014 and calculated the time between source and contact active TB diagnosis.

British Columbia is a Canadian province with 5.1 million people and an active TB incidence of 6.1 per 100 000 population [3]. The British Columbia Centre for Disease Control (BCCDC) TB registry houses diagnostic and treatment data for all individuals diagnosed with active TB in the province. Mandatory notification by public health partners and routine reporting from the centralized provincial mycobacteriology laboratory and provincial pharmacy make the TB registry virtually complete for all confirmed active TB cases. The BCCDC Public Health Laboratory (BCPHL) receives primary specimens and *Mtb* cultures for all active TB cases in the province. It performs routine MIRU-VNTR genotyping for each culture-positive case. The BCPHL also has WGS results for all MIRU-VNTR clustered *Mtb* isolates identified in BC between 2005 and 2014 [8].

For this study, we linked the BCCDC Provincial TB Registry and the BCPHL WGS data using Personal Health Numbers, which are unique lifetime identifiers assigned to BC residents. Contact investigation classifications were defined per the Canadian TB Standards and BCCDC TB manual [3, 9]. We defined a secondary TB transmission event as an *Mtb* culture-positive case whose *Mtb* genome sequence varied by 5 or fewer single nucleotide variants (SNVs) from another *Mtb* isolate with a known history of contact. This transmission threshold has been used previously to exclude cases that were not linked through recent transmission [10]. A contact was defined as a person who had been exposed to an active TB case and identified via contact investigations, as reported in the Provincial TB Registry. We classified contacts as either household, close nonhousehold, or casual contacts as per the BCCDC TB manual [9]. The time to development of secondary TB was calculated by subtracting the number of months from source diagnosis date to contact diagnosis date. Clusters of 5 or fewer people were removed to account for uncertain outbreak transmission patterns that cannot be inferred using the SNV-based threshold approach. For sensitivity analyses, we removed people diagnosed within 30 days of the source diagnosis and expanded to a 12-SNV threshold.

Linking the 2 data sets, we identified 37 secondary TB transmission events, attributed to 35 unique source cases with pulmonary TB (Table 1). Of the 37 people who developed secondary TB, 36 (97.3%) were diagnosed with pulmonary TB. The one individual who developed extrapulmonary TB was diagnosed with paraspinal TB. In total, 30 (81.0%) people with secondary TB were household contacts.

**Table 1.** Characteristics of People With Source and Secondary Tuberculosis

	Source (N = 35)	Secondary (N = 37)
Sex, n (%)		
Male	21 (60.0)	22 (59.5)
Female	14 (40.0)	15 (40.5)
Age, median (Q1, Q3)		
Years, at time of diagnosis	48.0 (37.0, 62.5)	40.0 (26.0, 49.0)
Country of birth, n (%)		
Foreign born	19 (54.3)	9 (24.3)
Canadian born	16 (45.7)	28 (75.7)
TB site, n (%)		
Pulmonary	35 (100.0)	36 (97.3)
Extrapulmonary	0 (0.0)	1 (2.7)
AFB smear, n (%)		
Positive^a^	33 (94.2)	22 (61.1)
Comorbidities, n (%)		
HIV coinfection	2 (5.7)	4 (10.8)
Diabetes	2 (5.7)	2 (5.4)
History of TB, n (%)		
Prior active TB diagnosis	1 (2.9)	1 (2.7)
Prior completion of latent TB treatment	1 (2.9)	3 (8.1)
Contact type, n (%)		
Household	…	30 (81.0)
Close	…	3 (8.1)
Casual	…	4 (10.8)

Abbreviations: AFB, acid-fast bacillus; HIV, human immunodeficiency virus; Q, quartile; TB, tuberculosis.

^a^For those with pulmonary TB.

Overall, the median time to development of secondary TB was 8.8 months (quartile [Q] 1, Q3: 2.6, 19.6 months) with a range from 8 days to 88.6 months (Figure 1). When we restricted the analysis to only household contacts, the median time to development of secondary TB was 9.5 months (Q1, Q3: 2.7, 18.9 months). Removing 3 source–contact dyads separated by less than 30 days increased the median time to the development of secondary TB to 11.0 months (Q1, Q3: 4.2, 26.4 months). Expanding to a 12-SNV threshold identified 1 further source–contact dyad separated by 27 days. Including the 12-SNV source–contact dyad, the median time to development of secondary TB was 8.6 months (Q1, Q3: 2.4, 18.9 months).

**Figure 1.**
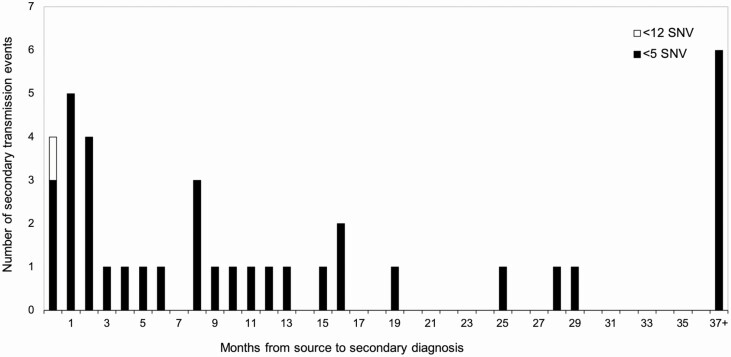
Frequency distribution of the number of secondary TB transmission events. Abbreviations: SNV, single nucleotide variant; TB, tuberculosis.

Although limited by sample size, our findings add to the growing body of evidence demonstrating that secondary TB among exposed contacts is highest in the first year after exposure and declines after that [4, 11]. In our study, 45% of the secondary transmission occurred within the first 6 months. Similar to our research, Borgdorff et al [12] used a combination of molecular techniques with epidemiological data to calculate the timing of secondary TB in the Netherlands. Results from this study indicate, in a highly selected group of source–contact dyads outside of an outbreak setting, that the median incubation period for secondary cases was 1.3 years, with probabilities of contact with active TB developing the disease within 1, 2, and 5 years of 45%, 62%, and 83%, respectively [12].

Our findings indicate that over 30% of secondary TB transmission events occurred within the first 3 months of identification of the source. These findings emphasize the importance of timely contact investigations and the relevance of rapid screening and initiation of preventative therapy The US Centers for Disease Control and Prevention recommend the source case be interviewed to identify contacts within 1 business day of being reported and that the initial assessment of high-priority contacts be within 1 week of identification, and completed within 1 month. However, due to the complex and multistep nature of contact investigations, the practical execution of a contact investigation can take anywhere from a few weeks to many months to complete. Reports from the United States and Canada estimate that the time from index case diagnosis to contact initiation of preventive therapy ranges from 53 days under study conditions to 270 days under programmatic conditions, highlighting the realities of the time requirements needed to carry out contact investigations [13, 14].

In conclusion, by linking WGS results to epidemiological data, we found the median time to development of secondary TB ranged between 8 to 11 months, with 30% occurring within the first 3 months. Strategies aimed at improving the timeliness of identifying and treating people recently exposed are likely to impact TB elimination strategies in low-TB-incidence regions such as BC.

## Note

***Potential conflicts of interest***. The authors: No reported conflicts of interest. All authors have submitted the ICMJE Form for Disclosure of Potential Conflicts of Interest.
